# Adaptive capacities for safe clinical practice for patients hospitalised during a suicidal crisis: a qualitative study

**DOI:** 10.1186/s12888-020-02689-8

**Published:** 2020-06-19

**Authors:** Siv Hilde Berg, Kristine Rørtveit, Fredrik A. Walby, Karina Aase

**Affiliations:** 1 Division of Adult Mental Health, Sandnes DPS, Stavanger University Hospital, Postveien 181, N-4307 Sandnes, Norway; 2 Nursing and Health Care Research Group, Department of Research, Stavanger University Hospital, P.O. Box 8100, N-4068 Stavanger, Norway; 3 National Centre for Suicide Research and Prevention, Faculty of Medicine, University of Oslo, Sognsvannsveien 21, Building 12, N-0320 Oslo, Norway; 4 Centre for Resilience in Healthcare, Faculty of Health Sciences, University of Stavanger, N-4036 Stavanger, Norway

**Keywords:** Adaptation, Sense making, Trade-offs, Mental health, Suicide, Uncertainty

## Abstract

**Background:**

Safe clinical practice for patients hospitalised in mental health care during a suicidal crisis is situated within a dynamic, non-linear and uncertain context. Under such complex conditions, the adaptive capacity is considered vital to handling challenges and changes in clinical care. This study aimed to explore safe clinical practice for suicidal patients hospitalised in mental health wards through understanding healthcare professionals’ (HCPs’) capacities to adapt to challenges and changes in clinical care.

**Methods:**

This study applied a qualitative design with focus group and individual interviews. Twenty-five HCPs participated in the focus groups, and 18 participated in individual interviews. The study was conducted in open and locked wards in a university hospital in Norway providing specialised mental health services for patients with mental illness.

**Results:**

HCPs described their adaptive capacities for clinical practice relative to three themes. 1) HCPs *used expertise to make sense of suicidal behaviour* to support complex decision making. Their strategies included setting aside forms and checklists to prioritise trust and making judgements based on more than just patients’ spoken words. They improved their understanding by seeking others’ perspectives through collaborative sense-making processes involving the healthcare team and patient. 2) HCPs *individualised the therapeutic milieu* to address the diversity of patients with suicidal behaviour by creating individual clinical pathways, making trade-offs between under- and over-protection and adjusting observations. 3) HCPs described *managing uncertainty* as necessary for providing safe clinical practice. They managed uncertainty as a team by developing mutual collegial trust and support and creating a shared understanding.

**Conclusion:**

HCPs’ adaptive capacities are vital to the complex set of practices involved in safe clinical practice for patients hospitalised during a suicidal crisis. By using expertise, individualising the therapeutic milieu, and managing uncertainty, HCPs individually and collectively develop their capacities to adapt to challenges and changes in clinical care. HCPs cannot easily ensure safe clinical practice by following standards; safe clinical practice depends on HCPs’ adaptations. Ward systems that ensure collegial trust and support, as well as arenas that foster shared understanding and situational awareness, are needed.

## Background

Suicide is a particular concern for patient safety in mental health wards. However, knowledge to support an understanding of safety for patients hospitalised during a suicidal crisis is lacking [1, 2]. Despite the growing body of literature on patient safety research, knowledge on patient safety in mental health settings is limited [3]. Studies have documented that different safety practices are simultaneously enacted in mental health care. The personalised- psychological safety and therapeutic safety are created in the personal contact with patients, and healthcare professionals (HCPs) attempt to manage risk by ensuring that suicidal patients feel safe [4–6]. Technical safety and disciplinary safety attempt to reduce risk through implementing barriers and control systems, such as physical infrastructure and the documentation of suicide risk [5–7].

Preventing suicides in wards is undoubtedly a complex and challenging task. It is well documented that HCPs who care for suicidal patients carry an emotional burden and experience fear of blame [8–11]. Clinical suicide risk instruments and risk scales do not enable HCPs to predict which patients will die by suicide [12–15], and clinical decision making in hospital wards often involves a high degree of uncertainty. Suicidal behaviour is characterised by aetiological heterogeneity both in terms of presentation and treatment [16]; thus, each patient needs to be understood and approached differently. In particular, being detected by mindful HCPs who show sensitivity toward the individual during acute suicidal deteriorations, receiving tailor-made treatment and being protected by adaptive practice are vital for suicidal patients’ experiences of safe clinical practice [17].

Safe clinical practice for a patient during a suicidal crisis is situated within a dynamic, non-linear and uncertain context [18, 19]. Under such complex conditions, the adaptive capacity is considered vital to handling challenges and changes in clinical care [20–22]. To ensure for good outcomes for patients HCPs make adaptations by relying on their skills, knowledge and experience [20], and they go beyond their assigned tasks and roles to adapt in everyday practice [23, 24]. Although adaptability is perceived as a source of safety in complex practices, it is acknowledged that adaptability may also have negative consequences [20, 25–27].

Studies of clinical decision making in complex care settings have found that HCPs constantly make trade-offs between competing goals, adjust procedures to complete their work, and apply sense-making skills to increase their situational awareness of ill-structured situations. These are all examples of adaptive capacities that HCPs exhibit in different healthcare contexts [27]. Such adaptive capacities also apply to suicide risk detection and response in clinical care practices. A study among community-based mental health workers in the UK revealed a complex decision-making process involving uncertainty and trade-offs regarding patient clinical needs, patient desires, legal and procedural obligations, and resource considerations [28].

What particularly distinguishes an expert from a novice is the ability to make sense of comprehensive and complex information through situational awareness [29]. These abilities are essential for adaptation [20]. Currently, there is a lack of literature regarding how HCPs use their expertise to improve clinical decision making in mental health [30], how they experience challenges and changes, and how they adapt to ensure safe clinical practice for patients hospitalised during a suicidal crisis. Inpatient care settings for suicidal patients involve clinical decision making about multiple aspects of safe care, e.g., acute and long-term risk management, physical protection and coordination of multi-professional care. This study aimed to explore safe clinical practice for suicidal patients hospitalised in mental health wards through understanding healthcare professionals’ (HCPs’) capacities to adapt to challenges and changes in clinical care. The specific research question was as follows: How can we describe the adaptive capacities that HCPs use to ensure safe clinical practice for patients hospitalised during a suicidal crisis?

## Methods

### Study context

The study was conducted at a university hospital in Norway that provides specialised mental health services for patients with mental illness. The hospital treats approximately 10,000 patients per year. A national patient safety programme for suicide prevention was ongoing in the hospital wards during data collection. This national programme included a checklist to document whether the patient had been assessed for suicide risk, had received an assessment by a specialist within the first day, and had received a safety plan and follow-up appointment at discharge and whether the next of kin had been contacted [31, 32]. In addition, the hospital had developed its own forms for documenting risk factors and warning signs for suicide risk. National guidelines for the prevention of suicide in mental health care systems were also implemented [33].

### Study design

The study applied a qualitative design with focus group interviews and individual interviews [34, 35]. The purpose of the use of multiple, complementary methods was to increase the understanding of the studied phenomenon of safe clinical practice [36, 37]. We applied sequential triangulation to integrate the data into a comprehensive whole, as described by Morse [38, 39]. First, we conducted focus group interviews to explore and identify the relevant values and perspectives on safe clinical practice. Then, we performed individual interviews to study in depth the themes that emerged in the focus group interviews [38, 39].

### Data collection

We employed a purposeful sampling strategy, aiming to recruit HCPs who were working in open or locked wards in specialised mental health care settings for adults and who had different levels of expertise and diverse professional backgrounds [40]. We recruited HCPs from nine wards. The locked wards specialised in psychosis (*n* = 1), affective disorders (n = 1) or acute care (*n* = 2), and the open wards specialised in rehabilitation (*n* = 3) or short-term stabilisation during crisis (n = 2). The sample included nurses (registered nurses with and without a specialisation in mental health), social educators, a social worker, medical doctors (physicians and consultant psychiatrists), and consultant clinical psychologists (with and without a specialisation in clinical adult psychology). The participants were of both genders (7 males and 28 women) and had one to 24 years of work experience in mental health wards. We considered participants who had one to two years of experience to be novices and those who had more than five years of experience and a specialisation in mental health to have a high level of expertise. A sample of sufficient size was needed to represent the variation among HCPs involved in the provision of safe clinical practice [41]. We prioritised the representation of variations in gender, expertise and experience, professional backgrounds, and open/locked wards in the sample [42]. We evaluated the sample size continuously during the research process and considered the final sample to provide adequate information power [41]. To be included in the focus group interviews and the individual interviews, HCPs had to voluntarily consent to participate. None of the participants dropped out of the study. The interviews took place at a location close to the HCPs’ workplace. The interviews were performed face to face and were audio-recorded. The researchers explained that the purpose of the study was to understand, not to evaluate, the participants’ practices. Data were collected from May to December 2016.

### Focus group interviews

Five focus group interviews were performed [34, 43], and a total of 25 HCPs from eight open and locked wards were included in the groups (Table 1). The interviews followed a semi-structured interview guide that was developed in collaboration with the advisory panel and pilot tested (additional file 1). Either SHB or KR moderated the interviews, and SHB or Marie Anbjørnsen co-moderated the interviews (see Acknowledgements). We made modifications to the interview guide after each interview to continuously improve the understanding of safe clinical practice in the mental health wards. During the interviews, we asked open-ended questions about experiences working with suicidal patients in wards, contingencies for good outcomes and safe clinical practice, and experiences with safety measures. The interviews lasted 90 min and yielded data about the participants’ emotions, opinions and challenges related to safe clinical practice.

**Table 1:** Participants in the focus group interviews

Group nr.	Participants	Setting
1. (pilot)	5 nurses	1 open ward
2.	2 psychologists, 4 medical doctors	3 locked wards
3.	3 psychologists, 2 medical doctors	2 open wards
4.	4 nurses	3 locked wards
5.	5 nurses	3 open wards

### Individual interviews

We conducted individual interviews [34] with 18 HCPs from seven mental health wards (Table 2). Eight of the participants had participated in a focus group interview, which allowed us to follow up on specific issues from the focus groups with some of the participants while also including participants who were not influenced by the focus group discussions and thus could provide more intuitive reflections. SHB conducted the individual interviews utilising a semi-structured interview guide that had been developed and pilot tested (additional file 1). The interview guide aimed to elicit participants’ elaboration on in-depth topics related to the five themes generated by the focus group interviews: making sense of suicidal behaviour, creating a shared understanding, handling emotional burdens, providing treatment and protection and learning from practice. The individual interviews lasted approximately 60 min and yielded data about each participant’s feelings, experiences and strategies.

**Table 2:** Participants in the individual interviews

Participants	Setting
3 psychologists	1 locked ward and 2 open wards
4 medical doctors	1 locked ward and 1 open ward
11 nurses	2 locked wards and 3 open wards

### Data analysis

We analysed the data material from the focus group interviews and individual interviews sequentially [38] using Graneheim and Lundman’s method for qualitative content analysis [44]. Consistent with a phenomenological hermeneutic point of view, we aimed to be open to the meanings presented by the participants and the relationships between the parts and the whole [34]. The analysis involved systematic movement from the manifest content towards a higher level of abstraction and interpretation, as well as movement back and forth between the content and interpretation to elicit meaning [45]. SHB read each interview transcript to gain an overall understanding of the participants’ expressions. KR and KAA read a selection of the interviews and collaborated in discussions of their first impressions. SHB marked and condensed meaning units, generated codes that represented the manifest content, and developed categories across the data set that unified the codes. In the next stage, SHB sorted the categories into content areas and then abstracted them into sub-themes and themes. All authors collaborated analytically in the generation of themes. Finally, we triangulated the results from the focus groups and the individual interviews to generate integrated sub-themes and themes [34]. The integration of the data provided a more comprehensive picture and a fuller understanding than we could have been achieved by analysing the data collected with each method individually [38].

## Results

In the analysis, we identified a set of eight sub-themes and organised them into three major themes, each representing an adaptive capacity for safe clinical practice, as displayed in Table 3.

**Table 3:** Themes and sub-themes derived from the focus group and individual interviews

Themes	Sub-themes
Using expertise to make sense of suicidal behaviour	Setting aside the forms and checklists to prioritise trust Making judgement based on more than patients’ spoken words Improving understanding by seeking others’ perspectives
Individualising the therapeutic milieu	Creating individual clinical pathways Making trade-offs between under- and over-protection Adjusting observations
Managing uncertainty	Building mutual collegial trust and support Creating a shared understanding

### Using expertise to make sense of suicidal behaviour

HCPs described their use of expertise to make sense of suicidal behaviour during risk assessment. They accomplished this by setting aside forms and checklist aside to prioritise trust, making judgements based on more than just patients’ spoken words, and improving their understanding by seeking others’ perspectives.

#### Setting aside the forms and checklist to prioritise trust

The participants emphasised the importance of establishing a trusting bond with patients during suicide risk assessment. They created a safe atmosphere and a trusting bond by engaging in a dialogue with the patient about his or her situation as a whole and by asking about suicidal ideations as a normal part of the dialogue. A female medical doctor described these practices as follows:

*“I start off easy and ask why they are here, and the more the patient talks about their challenges, the more you can go into the things he talks about, and then in a way, it leads to a natural transition to ‘when you have this struggle that you describe, have you ever had thoughts that it would have been easier to die or thoughts of taking your own life?’ I try to make a natural transition and create some trust during the conversation so the patient feels it’s safe to open up and talk about things along the way”* (1 year of experience, locked wards).

HCPs ensured that employing checklists and forms did not compromise the therapeutic relationship. Thus, they completed the checklist and the form for suicide risk assessments after talking with the patient. As patients opened up about their emotions, HCPs affirmed their feelings and approached them with non-judgemental and exploratory attitudes, providing hope and signalling that they were able to and had time to listen. They considered trust to be essential to obtaining honest answers. Through relational contact with patients, the HCPs made sense of patients’ spoken words and their individual ways of behaving and thinking when suicidal.

#### Making judgements based on more than patients’ spoken words

HCPs knew they could not always trust what patients reported and often paid attention to their “gut feelings”. They described the “gut feeling” as an unpleasant sense of uncertainty that made them worry that a patient was at immediate risk of suicide. The “gut feeling” was something they felt but could not express verbally, as described by a female medical doctor:

*“It’s often a gut feeling you get, and that is what makes it difficult. You should be able to document this in a suicide risk assessment. But it is, in a way, what happens in a meeting with the patient, their spoken and unspoken words, their background, their history, everything, in a way, the overall picture”* (1 year of experience, locked wards).

HCPs described their decision to trust a “gut feeling” as depending on the level of expertise and the quality of the therapeutic relationship with the patient. The experience of a “gut feeling” varied across situations and was related to a) a lack of contact and connection (e.g., lack of eye contact, withdrawal, lack of communication about suicidal ideations, poor mental state and/or lack of trust); b) a mismatch between a patient’s observed behaviour and his or her spoken words (e.g., saying she felt fine while showing signs of withdrawal and stress); and c) an unpredictable or sudden change in behaviour (e.g., acting drugged, agitated, or withdrawn or exhibiting sudden contempt or happiness):

*“Something happens with us when there are patients whom we are not familiar with; its feels more uncertain. Knowing how to ensure their safety is more challenging. We don’t know their signals and cues; we don’t know what we can use to keep them alive during crisis…. we don’t have the connection”* (Female social educator, 11 years of experience, open rehabilitation ward).

However, HCPs noted that they did not base their judgement solely on the “gut feeling”. Experienced medical doctors and psychologists described looking at the whole picture when trying to understand each patient’s suicidality and considering multiple sources of information. Experience increased the complexity of the information sources that were taken into account to understand the overall picture. Triangulating multiple sources of information improved HCPs’ situational awareness. Looking at the whole involved everything from considering the observed behaviour and what the patient did not report, including their ability to connect and make eye contact, to reviewing their previous medical history and mental health diagnosis.

The experienced HCPs felt that the checklist could not help them during assessment because it did not account for the information obtained from observing the patient’s behaviour, warning signs and current mental state. The novice HCPs preferred to follow the formal procedures and relied on risk factors, information from the patient’s medical journal, and the patient’s spoken words to assess suicide risk. They felt that the forms and the checklist helped them remember what to ask about.

HCPs perceived the “gut feeling” as fallible, as some had experienced patient suicide during inpatient care without sensing anything in advance. A male nurse described his thoughts after a young patient with schizophrenia died by suicide on leave from the ward:

*“He was a man of few words; he kept mostly to himself and did not talk about his emotions. He was hard to get through to, but few HCPs in the wards had a gut feeling that he was feeling so much pain. In the aftermath, we could see some warning sings, but no one anticipated it happening”* (4 years of experience, open rehabilitation ward).

While the medical doctors’ and psychologists’ suicide risk assessments were often restricted to consultations in a consultation room, the nurses’ practices were not temporally or spatially restricted. A female mental health nurse described the lack of restrictions as follows:

*“I can feel it just by being with them, and many times, especially if I know the patient, I can feel it before they can express it with words… She can tell me to leave and say everything is fine, and I will tell her that I feel I don’t want to leave you; I will stay. And often, after a while, she can explain she had suicide plans at that moment”* (24 years of experience, open rehabilitation ward).

The nurses were constantly alert to changes in suicidal behaviour.

#### Improving understanding by seeking others’ perspectives

HCPs improved their understanding of suicide risk by discussing cases with more experienced colleagues, their teams or professionals with other backgrounds.

*“We always talk with the patient together when assessing suicide. Then, we are two persons who can calibrate each other’s experience afterwards, to talk about it and assess the risk together”* (female nurse, 1.5 years of experience, short-term stabilisation ward).

Some HCPs reflected on their subjective clinical judgements together with the patient to make sense of the patient’s suicide risk. This strategy improved their situational awareness.

In particular, HCPs made difficult decisions, such as whether a suicidal patient was ready for reduced protection, in collaboration with their colleagues and the patient. However, they experienced that attempting to understand patients’ states of mind required face-to-face contact with them. Thus, there was limited value in consulting with the on-call doctors, as they had not seen the patients face to face and considered only the information they were given.

### Individualising the therapeutic milieu

HCPs described individualising the therapeutic milieu for the delivery of safe clinical practice. They achieved such individualisation by developing individualised clinical pathways, making trade-offs between under- and over-protection and making adjustments to be watchful and connected to provide protection.

#### Creating individual clinical pathways

HCPs considered suicidal patients to be a heterogeneous group: they believed there was no such thing as a typical “suicidal patient”. Safe clinical practice for these patients was therefore dependent on HCPs’ diverse approaches to the individual patients. An HCP’s approach to creating individual clinical pathways varied according to his or her professional perspective. The nurses emphasised the importance of patient involvement for the re-establishment of a sense of hope and dignity for the individual patient. The medical doctors emphasised the importance of individualised approaches that addressed underlying mental health disorders. The psychologists emphasised the need to explore what suicidality meant for each individual, the feelings behind the suicidal behaviour, the patient’s logic, the patient’s despair, and unique warning signs and triggers. A psychologist explained how he helped patients feel safe from suicide by helping them gain insight and emotional control:

“*I work with the individual patients’ underlying feelings about suicidality… Through gaining insight, the patients find other ways to express their emotions”* (male psychologist with specialisation, 15 years of experience, open rehabilitation ward).

The therapeutic milieu had a calming effect on patients with “chaotic and acute suicidal behaviour” through its daily structure for activities, rest and meals. However, to ensure safe clinical practice for the patients, HCPs needed the flexibility to individualise the therapeutic milieu within the frames of this predictable structure, which again depended on their expertise:

*“You need good people who have the expertise to interact with people; without that, you won’t benefit from any structure, systems or forms to fill out”* (female consultant psychiatrist, 10 years of experience, locked ward).

Individualised approaches were considered essential for making a safety plan. However, safety planning did not always emphasise individualisation. All patients were offered a safety plan consisting of a list of individual warning signs, coping strategies, and sources of support. To make these plans effective for patient safety, HCPs co-created them with the patient so that they reflected the patient’s conditions and coping strategies. Development of the safety plan was dependent on the therapeutic relationship with the patient and the patient’s capability to reflect and gain insight. The creation of the plan sometimes was delayed due to the patient’s mental condition and other times was delayed because HCPs were overloaded with discharge tasks. The safety procedure focused on documenting whether a plan had been created. Thus, HCPs often hastily created a plan without patient engagement just to “get the job done”. Without individualisation, the safety plan lost its function as a safety tool for the patient, as it was not actively used during a crisis:

*“The safety plan, it’s stressful. We must start early to make it count, but sometimes, I see patients standing in the hallway with their luggage ready for discharge, and a stressed nurse runs after them and says, ‘Wait, this is a safety plan’”* (nurse, 8 years of experience, locked ward).

HCPs considered therapeutic and individualised approaches to be essential in conversations about suicidal ideations; however, the procedures focused merely on documenting suicide risk assessment and not on how to talk about suicide. Medical doctors and psychologists were supposed to complete a form and a checklist for suicide risk assessment to ensure that risk factors were taken into account. HCPs described competing goals: documenting risk vs. approaching patients’ feelings and understanding them as individuals. Their strategy to achieve safe clinical practice was to prioritise the therapeutic conversation with the patient, eliminating questions about risk factors that they considered irrelevant. They completed the forms and checklist for suicide risk assessments after talking with the patient.

#### Making trade-offs between under- and over-protection

HCPs’ considered making judgements about a safe level of protection to be a difficult and complex task. Making these judgements was a dynamic process that required constant monitoring of the level of suicide risk and continuous adjustments to the level of protection. A safe level of protection also depended on the individual patient’s underlying mental health problems and therefore needed to be individualised. A female consultant psychiatrist illustrated the complexity of the task:

*“I feel damned if I do and damned if I don’t. Society criticises us (specialised mental health care) for using too many physical constraints and calls for more autonomy (for the patient), but at the same time, we are made accountable for the suicides and are told that we should have done more to prevent them. They (members of society) don’t truly comprehend the complexity of this task”* (10 years of experience, locked ward).

To cope with this complexity, HCPs made trade-offs between under-protection and over-protection for each patient. They used some rough categories to distinguish suicidal patients’ need for protection. They often categorised patients with affective disorders and psychotic disorders as “acute suicidal” and categorised patients who constantly struggled with suicidal ideations and often used suicidality as an approach to communicate hopelessness or a need for something else as “chronic suicidal”. For participants with acute suicidal behaviour, the greatest fear among HCPs was the under-protection of the patient (e.g., access to lethal means when hospitalised in an open ward), particularly during psychotic phases. In these cases, HCPs prioritised physically preventing the patient from attempting suicide, with the risk of over-protection (e.g., loss of autonomy).

HCPs perceived both under-protecting and over-protecting (e.g., constant observation for a long period) patients with chronic suicidal behaviour to be harmful. Therefore, the HCPs constantly assessed patients’ suicidality and made daily trade-offs. They had to decide whether to empower the patient to take responsibility for his/her own safety, despite the risk of suicide attempts, or to increase protection for a brief period at the risk of worsening the suicide risk and reducing the patient’s sense of independence. A nurse described the complexity of making such judgements in daily practices:

“*If there is a chronic suicidal patient, one should not talk about suicidality all the time. Therefore, I don’t want to ask if the patient has thoughts of suicide before I let that patient out unless the patient says very clearly that he or she has suicidal plans. If I see that the patient struggles, I would ask the patient, ‘Do you think it is okay for you to go out now?’, and then you will get some gut feeling about this. It has been difficult at times to risk locking out patients, especially at night and on weekends, when you are alone there. However, there is an assessment the therapist has done, and we have to stick to the plan, especially with emotionally unstable patients with chronic suicidality. You have to give them responsibility back, and it is challenging”* (female mental health nurse, 4 years of experience, locked acute ward).

Safe clinical practice for patients with chronic suicidal behaviour involves a delicate balance between under- and over-protection.

#### Adjusting observation

Although the procedures distinguished between constant and intermittent observation with specified intervals, HCPs reported taking individualised approaches to ensure patient safety with multiple considerations: they aimed to ensure connection with the patient without neglecting the need to be watchful and to take the patient’s need for privacy into account while still physically protecting him or her from a suicide attempt. HCPs noted that all patients had their own ways of connecting and feeling safe, for example, some patients wanted to talk, while others just needed to be assured that HCPs were present if they needed them:

*“I understood that he had a desire to talk, but then there is almost a kind of rejection when you go out again. Then, you come back again after 5 minutes, look in and go out again. It’s like, ‘I just have to check that you are still alive’; it’s not an act of kindness. I always try to get them out of the room, so it becomes less forced, and I can give more attention to them”* (male nurse, 1 year of experience, locked ward).

Keeping patients safe during observation involved making adjustments in observations of the individual and finding ways to re-establish the patient’s sense of dignity while still being watchful.

### Managing uncertainty

HCPs described managing personal uncertainty by building mutual trust and support and a creating a shared understanding of safe practice.

#### Building mutual collegial trust and support

HCPs felt constantly alert and worried about suicide in their daily work, despite knowing that such incidents rarely happen. They often left work with a feeling of uncertainty, and they knew that when caring for suicidal patients, it was not possible to be 100% certain that the patient would not die by suicide. Furthermore, HCPs often felt driven by a fear of being held accountable for a suicide and being responsible for an adverse event. They applied strategies to avoid blame and responsibility in the case of a suicide, such as excessively documenting information in the patient’s journal, ensuring that someone else was involved in decisions about suicide risk or simply transferring responsibility for the patient to someone else or to another ward. They perceived these strategies as threats to patient safety because they compromised HCPs’ ability to fulfil patients’ therapeutic needs. HCPs felt that their focus shifted from doing their best for the patient to making sure they were “covering their backs”. HCPs believed that they lacked the agency to address these issues, which elicited feelings of hopelessness and shame.

To address uncertainty, HCPs needed a climate of mutual trust and support in which they felt safe enough to be vulnerable and unsure. Such a climate allowed them to discuss their doubts and uncertainties with the ability to express disagreement within the team while also taking a coordinated approach to caring for the patient, as described by a male nurse:

*“If we have good communication within the team, we will be able to spread it and take a safe approach to the patients. However, if we have a poor climate in the ward, it will reflect on the patients. There will be disagreements and aggression”* (1 year of experience, locked ward).

Managing uncertainty also involved doing the right thing and not having to complete difficult tasks alone. Thus, when the ward supported patient-centred care and provided arenas for support, case reflection and learning, HCPs were able to address the emotional burden of caring for suicidal patients.

Support mechanisms needed to be adaptive and support ad hoc responses to immediate needs for feedback. After suicides and suicide attempts, HCPs needed to be assured that they would not be used as a scapegoat by the clinical team or the organisation as a whole. In this context, leadership support and team debriefings were perceived as important. While psychologists and medical doctors described multiple structures for support, nurses often described lacking formal support systems in the wards.

*“Many times, you don’t want to open that door alone. You never know what you will find behind that door, so you go together in pairs. It is safe to have someone with you because many times when you enter, they (patients) have tried to strangle themselves or cut their wrists... It’s an emotional burden to find them in all these situations”* (female social educator, 3 years of experience, locked ward).

The nurses self-organised and conducted nurse observations together to ensure they did not carry the burden alone. Instead of receiving formal supervision, they had informal conversations after work. They supported each other by making difficult decisions together to avoid one person becoming the scapegoat for adverse events.

#### Creating a shared understanding

Considering how to approach suicidal behaviour often generated feelings of uncertainty. HCPs had different understandings of suicidality and often disagreed on how to approach it. These disagreements were often related to determining the safe level of protection for patients in acute phases. In particular, patients often talked about their suicidality differently with different HCPs. In addition, nurses, psychologists and medical doctors had different tasks, responsibilities, and degrees of familiarity and therapeutic relationships with the patient, which affected their perceptions of risk and the acceptable level of uncertainty for each patient. Safe clinical practice for suicidal patients was seen as dependent on reducing uncertainty through feeling capable in his or her professional role.

Diverse approaches influenced by different psychotherapeutic schools served to create common ground in three of the nine wards included in this study. By applying the same therapeutic approach, all the professional groups shared multiple arenas for training, supervision, and education using the same patient-directed tools and language. Having common ground helped them approach the patient as a team.

## Discussion

The current study documents three main adaptive capacities used by HCPs to provide safe clinical practice for patients in mental health wards during a suicidal crisis.

### Using expertise

The theme *using expertise to make sense of suicidal behaviour* describes an adaptive capacity involving strategies to deal with uncertainty. The findings indicate that experts use intuition and detect warning signs for suicidal behaviour to make sense of uncertainty and to manage complex and high-risk decision making [46]. This finding corresponds with a previous study by Waern et al. [47] that found that few HCPs used checklists but translated non-verbal cues into a “gut feeling”, which was essential to the assessment process. This study adds to the knowledge that intuition is not the sole source of information in an assessment; rather, it supplements multiple sources of context-specific and general information, which together improve situational awareness [48].

The findings also reflect the importance of collaborative sense-making processes and the improvement of expertise through teamwork. As such, there is a need to directly support the creation of shared situational awareness that involves both healthcare teams and patients. Training in suicide risk assessment can benefit multidisciplinary training for HCPs who regularly interact as a team to establish a shared vision and values [49, 50]. In addition, training can benefit from the use of real-life examples of clinical decision making [51] and educating HCPs in collaborative approaches to suicide risk assessment that involve patient perspectives [52, 53].

Consistent with studies on expertise, this study indicates that novice HCPs focus on patients’ verbal reports, written information in patients’ journals, and formal risk factors, while experienced HCPs rely more on non-verbal information, cues and their intuition to understand what constitutes critical suicide behaviour [20, 54]. This finding suggests that HCPs require different guidance at different stages of expertise development, which is in accordance with the findings of Benner et al. [55, 56]. These authors claimed that novice HCPs need context-free rules to guide their task performance, while experts’ decision making cannot be captured in explicit formal steps because they no longer use rules to guide their practice; instead, they use past concrete experiences [55, 56]. However, a fallacy is that the novice HCP may be over-focused on rules at the expense of being insensitive to the context and the individual patient, while the expert HCP may be overconfident, relying on intuition and taking pride in risk-taking [20]. Working together as a team to make sense of suicidal behaviour improves HCPs’ comprehension and interpretation of the information obtained and might thus improve situational awareness for both novice and expert HCPs [57].

### Individualising the therapeutic milieu

The theme *individualising the therapeutic milieu* describes both an adaptive capacity and conditions in which adaptations are vital to ensure safe clinical practice.

A number of studies have shown that patients experience constant observation as non-therapeutic due to, e.g., the lack of acknowledgement, lack of privacy and lack of empathy [58–62]. Studies have reported the importance of having experienced staff [63] who are therapeutically engaged with the patient [58] and interchange between exerting control and building the therapeutic relationship during constant observation [64]. The findings reflect that HCPs cope with the complexity of safe protection by making trade-offs between higher- and lower-level goals [65] and by ensuring that protection is individualised by taking multiple considerations into account for each patient through adaptations [66]. Guideline development should acknowledge the expertise needed to provide protection safely for each individual.

In accordance with the literature, the finding reflects that patient involvement and the individualisation of safety plans [67] and suicide risk assessments [52, 68] are essential for effective safe clinical practice. HCPs in this study described their attempts to involve the patient in and individualise the safety plan, but they did not always succeed. The intention of a safety plan is to help patients cope with symptoms at an early stage, and interventions emphasise patient involvement [67]. These prerequisites might not have been communicated properly when the checklist was introduced as part of the national patient safety programme for suicide prevention [31, 32]. Some patients might also be in a mental state that hinders them from being involved in making their own safety plan.

The findings imply that therapeutic measures and safety measures are not necessarily separate entities that are driven by distinct logics: they rely on individualisation and the therapeutic relationship. Efforts to better integrate safe clinical care across the technical-disciplinary perspective and the therapeutic and individualised perspective [5, 10] may benefit from the development of expertise in suicide risk assessment, constant observation, and the creation of safety plans, as well as requirements for documenting practice.

### Managing uncertainty

The theme *managing uncertainty* describes an adaptive capacity that corresponds with the findings of previous studies that caring for suicidal patients involves dealing with uncertainty [8, 9, 69]. The findings reflect that ward systems that ensure mutual trust and support and a shared understanding help HCPs deal with the uncertainty surrounding suicide risk and provide essential support for safe clinical practice. Having common ground is related to the development of shared mental models and shared situational awareness in teams [70, 71], which is a strategy to reduce uncertainty in ambiguous situations by making HCPs able to improve their comprehension of the situation [72]. Furthermore, as uncertainty is managed through mutual collegial trust and collegial support, there is a need to create systems that ensure feedback on safe clinical practice and to foster HCPs’ trust that their colleagues will provide constructive support [20, 73]. The findings also reflect that a lack of support systems to address uncertainty can lead to the emergence of counterproductive behaviour among HCPs to protect themselves from punishment. These findings support Undrill’s [7] arguments that unintended consequences may arise in suicide prevention if HCPs are put in a position in which they feel a greater need to protect themselves than to protect patients. This study finds that to counteract such mechanisms, HCPs must address uncertainty as a team, and management responsibility should be emphasised through the establishment of formal support structures in wards. These findings correspond with previous study findings that nurses often call for formal support arenas [9, 74], supervision and training when caring for suicidal patients [8, 60]. These formal support arenas are often guaranteed for psychologists during their specialisation in clinical adult psychology and for medical doctors during their specialisation in psychiatry. This study reflects the importance of support structures for all professional groups to achieve safe clinical practice. Relying too heavily on individual HCPs’ capacities to adapt without providing support to maintain these capacities will eventually cause overload and burnout and leave the system brittle to adverse events [21, 27].

### Strengths and limitations

This study applied two different data collection methods to develop a comprehensive understanding of safe clinical practice. Multiple researchers participated in the data collection and analysis, adding various perspectives and breadth to the study of the phenomenon of interest [36]. We did not conduct member checks; instead, the advisory panel and co-authors helped test the coherence and plausibility of the interpretations [34]. The use of triangulation and the variety of the study settings strengthened the internal validity of the study. The external validity of the study is limited, as we conducted the study within a single hospital, and the local organisational culture therefore affected the study. However, the study findings support analytical generalisations regarding safe clinical practice for patients hospitalised during a suicidal crisis [75]. The focus of this study was limited to safe clinical practice at the micro level within hospital ward settings. Researchers could gain increased insight into adaptive capacities by applying a meso-macro perspective (e.g., hospital management, government and regulators) to study adaptive capacities at the interface between primary and secondary care and by employing multiple methods, particularly direct observation of HCP interactions and strategies.

## Conclusions

HCPs’ adaptive capacities are a vital component of the complex set of practices involved in safe clinical practice for patients hospitalised during a suicidal crisis. By using expertise, individualising the therapeutic milieu, and managing uncertainty, HCPs develop their capacity to adapt to challenges and changes in clinical care, both individually and collectively. HCPs cannot easily ensure safe clinical practice simply by following standards; safe clinical practice depends on HCP adaptations. However, individual HCPs cannot hold the responsibility for safe clinical practice alone. Ward systems that ensure collegial trust and support, as well as arenas that support shared understanding and shared situational awareness, are needed.

## Supplementary information

**Additional file 1.** Guides for interviewing the healthcare professionals.

## Data Availability

The data generated and analysed during the current study are not publicly available to protect the anonymity of the participants. Materials may be available from the corresponding author upon reasonable request.

## Abbreviation

HCP: Healthcare professional
