# Supplementary material for: Adaptive capacities for safe clinical practice for patients hospitalised during a suicidal crisis: a qualitative study
Source: BMC Psychiatry. 2020 Jun 19;20:316. doi: 10.1186/s12888-020-02689-8 (PMC7304097; doi:10.1186/s12888-020-02689-8)
Supplement: Supplementary file 1 — Additional file 1. Guides for interviewing the healthcare professionals. [file 12888_2020_2689_MOESM1_ESM.docx]

## **Additional file 1: Guides for interviewing the health care professionals**

## **Focus groups**

**Opening question**

1. How do you experience working with suicidal patients?
   - How to you cope with the challenges?

**Contingencies for good outcomes**

1. What ensures good outcomes for suicidal patients during hospitalization?
   - at system, ward, individual levels
   - any harmful conditions?
2. What does the suicidal patient need during hospitalization to ensure good outcomes?
   - What do you do to ensure good outcomes?

**Patient safety and contingencies for safe care**

1. How do you experience implementing the measures in the patient safety campaign?
   - Which safety measures ensure safe care for suicidal patients?
   - Which do not?
   - What do you do to ensure safe care?
2. Do you think there is something else that should be included in the safety procedures?
   - What else is of importance for safe care?
   - What would be the ideal patient safety campaign?

**Individual interviews**

**Making sense of suicidal behaviour**

1. What characterizes patients who are hospitalized with suicidal behaviour in the ward?
2. What challenges do you encounter when working with suicidal patients?
   1. How do you solve these?
3. How do you assess suicide risk?
   1. What do you do to make the assessment useful?
   2. How do you identify whether the patient is acutely suicidal/deteriorating?
   3. What do you do to cope with it?

**Providing treatment and protection**

1. What’s in place in the ward when you experience good patient care for suicidal patients?
2. What are the contingencies for a good conversation about suicidality?
   1. When and where do you have these conversations with the patient?
3. What do you do to ensure good discharge processes?

**Creating shared understanding**

1. How do you work across different professional groups with suicidal patients?
   1. What arenas are of importance? Explain the function of the arena.
   2. What challenges arise? How are these solved?
   3. How do you ensure shared understanding?

**Handling emotional burden**

1. How do you experience uncertainty in encounters with suicidal patients?
   1. What is the uncertainty related to?
   2. How do you cope with the uncertainty?
2. How do you need to be taken care of on a daily basis when working with suicidal patients?

**Learning from practice**

1. How do you learn from good patient care?
   1. When and where does the learning take place?
